# Imaging-to-recanalization delay influences perfusion CT threshold calibration for follow-up infarct volume estimation

**DOI:** 10.1016/j.ejro.2026.100779

**Published:** 2026-06-18

**Authors:** Simo Karhi, Erik Palm, Mikko Taina, Pekka Jäkälä, Hannu Manninen, Juhana Hakumäki, Ritva Vanninen

**Affiliations:** a Department of Clinical Radiology, Kuopio University Hospital, Kuopio, Finland; b Unit of Radiology, Institute of Clinical Medicine, University of Eastern Finland, Kuopio Finland; c Unit of Neurology, Institute of Clinical Medicine, University of Eastern Finland, Kuopio, Finland; d Neuro Center, Kuopio University Hospital, Kuopio, Finland

**Keywords:** Thrombectomy, Perfusion Imaging, Computed Tomography, Middle Cerebral Artery, Ischemic Stroke

## Abstract

**Background and purpose:**

Computed tomography perfusion (CTP) imaging is used in acute ischemic stroke (AIS) to estimate admission ischemic core volume (ICV). This retrospective single-center study evaluated how relative cerebral blood flow (rCBF) thresholds applied within a single postprocessing software package (syngo.via Neuro Perfusion) relate to follow-up infarct volume (FIV), with emphasis on the effect of imaging-to-recanalization delay.

**Patients and methods:**

Among 555 consecutive patients undergoing endovascular treatment (EVT) for AIS between January 2016 and October 2021, 236 patients with middle cerebral artery or internal carotid artery occlusion were included. Admission ICV was calculated using rCBF thresholds from < 10% to < 30% in 2% increments and compared with FIV on postprocedural non-enhanced CT. Patients achieving complete reperfusion were further analyzed using cumulative 10-minute delay windows from ≤ 90 to ≤ 130 min.

**Results:**

Complete reperfusion was achieved in 102 patients. Among patients achieving complete reperfusion within ≤ 100 min (n = 43), the highest observed correlation between admission CTP-derived ICV and FIV was seen at rCBF thresholds of < 22% and < 20% (R=0.86 for both). Between these tied thresholds, rCBF < 20% showed less frequent and smaller-volume ICV overestimation. In the broader subgroup with complete reperfusion within ≤ 120 min (n = 64), the highest observed correlation was seen at rCBF < 24% (R=0.84).

**Conclusions:**

In this vendor-specific study, the best-correlating admission CTP-derived ischemic core threshold relative to follow-up infarct volume varied with imaging-to-recanalization delay. This pattern may be relevant when interpreting perfusion-derived core estimates in acute stroke workflows with variable non-avoidable treatment delays, but should be considered exploratory.

## 1 Introduction

Endovascular thrombectomy (EVT) using modern stent-retriever devices and multimodal imaging-based patient selection has been shown to be effective for the treatment of anterior-circulation large-vessel-occlusion acute ischemic stroke (AIS) in multiple randomized controlled trials [1], [2], [3], [4], [5], [6]. Subsequent meta-analyses have shown that EVT benefits most eligible patients, irrespective of their baseline or background characteristics [7]. In addition to non-enhanced computed tomography (NECT) and computed tomography angiography (CTA), software-assisted assessment of ischemic core volume (ICV) and hypoperfused tissue using computed tomography perfusion (CTP) or magnetic resonance imaging (MRI) is considered useful in selected patients presenting within 6–24 h from symptom onset, both to support treatment selection and to contextualize expected infarct evolution on follow-up imaging [8], [9], [10]. Although early trials used ICV thresholds such as ≥ 70 mL as exclusion criteria for EVT, subsequent studies have shown that admission CTP-derived core estimates may substantially overestimate eventual follow-up infarct volume (FIV) in some patients, particularly in the early time window [4], [5], [11], [12], [13]. Despite the procedural risk of symptomatic intracranial hemorrhage, recent large trials and a meta-analysis comparing functional outcomes between EVT and medical management alone established the benefit of thrombectomy in patients with larger infarct cores and with mismatch profiles that were earlier considered unfavorable, supporting more inclusive selection approaches across patient subgroups [14], [15], [16]. With the projected annual demand for mechanical thrombectomy rising, the development of even more accurate imaging systems to improve patient safety and optimize resource allocation has become increasingly important [17].

The use of automated ICV detection is largely based on trials that employed the threshold-driven RAPID Software (iSchemaView, Menlo Park, CA, USA), which applies a delay-insensitive deconvolution method for CTP analysis [3], [4], [8], [9], [10]. Alternative commercial solutions for ischemic core and penumbral volume quantification are now available. Suggested threshold configurations vary by vendor, which initially led to discrepant results when RAPID was compared with syngo.via Neuro Perfusion software (Siemens Healthcare GmbH, Erlangen, Germany) or the IntelliSpace Portal CT Brain Perfusion Package (Philips Healthcare, The Netherlands) [18]. Such inconsistencies are undesirable, as exaggerated or understated ICV estimates on admission imaging may affect treatment interpretation and prognostic evaluation.

Relative cerebral blood flow (rCBF), expressed as CBF relative to reference normally perfused brain tissue, is widely used for automated ischemic core estimation, although reported threshold levels vary substantially across post-processing platforms [19], [20], [21], [22]. RAPID Software classifies regions with rCBF < 30% as ischemic core by default and uses time-to-maximum (Tmax) > 6 s to define hypoperfused tissue [4]. However, the direct application of these parameters in the syngo.via or IntelliSpace Portal software produced ICV estimates that were significantly larger than those generated with RAPID [23]. Further studies showed that adjusting the rCBF threshold to < 20% reduced disagreement between RAPID and syngo.via and yielded more comparable volumetric estimates; this threshold is now recommended by the manufacturer as the default setting for the Siemens application [24], [25].

A more comprehensive assessment of perfusion parameters is needed to refine patient selection for EVT and to facilitate comparisons across study populations. Because admission CTP-derived ischemic core estimates and follow-up infarct volumes are related but not interchangeable, threshold performance should be interpreted in the context of treatment timing. In this retrospective single-center study, we examined how vendor-specific admission CT perfusion–derived ICV thresholds generated with syngo.via relate to FIV across different imaging-to-recanalization delays. Threshold performance was defined using a hierarchical approach: within each imaging-to-recanalization delay window, the primary criterion was the highest observed correlation between admission ICV and FIV; when adjacent thresholds showed similar correlations, preference was given to thresholds with less frequent and smaller-volume ICV overestimation. This analysis sought to clarify earlier findings in a consecutive Finnish anterior-circulation AIS cohort undergoing EVT, with emphasis on patients achieving rapid complete reperfusion.

## 2 Materials and methods

### 2.1 Patients

The study was conducted according to the principles of the Declaration of Helsinki and was approved by the Research Ethics Board of Kuopio University Hospital (no. 5772789, 12 March 2013). The requirement for informed consent was waived due to the retrospective nature of the study and the use of routinely collected clinical data. Data were analyzed in pseudonymized form in accordance with applicable hospital guidelines and relevant data protection laws and regulations. In our tertiary center, all patients with acute ischemic stroke (AIS) between January 2016 and October 2021 (n ≈ 4070) underwent a diagnostic stroke imaging protocol, including NECT and CTA, with complementary CTP performed on admission when clinically appropriate. During this period, 555 patients aged ≥ 18 years underwent EVT after selection based on clot location, symptom duration, treatment delay, pre-stroke functional status, and overall clinical condition.

Out of the 555 consecutive patients undergoing EVT, 236 patients (mean age 70.2 years, 115 female) with anterior-circulation large-artery occlusion of the internal carotid artery (ICA, n = 37) or middle cerebral artery (MCA, n = 199; segments M1, n = 117; M2, n = 76; and M3, n = 6) were included in the study. Medical history including underlying coronary artery disease, hypertension, hypercholesterolemia, known atrial fibrillation, heart failure, and diabetes, as well as diagnostic imaging delays, were retrospectively collected from patient records. The baseline and clinical characteristics of the study population are summarized in Table 1**.**

**Table 1:** Patient baseline information.

**Baseline characteristic**	**n**	**%**
Included patients, total	236	100
- Female	115	48.7
- Male	121	51.3
Age, years (mean 70.2 ± 11.6 SD)		
- Age over 80 years	44	18.6
Earlier stroke or intracerebral hemorrhage	57	24.1
- Ischemic Stroke or TIA	45	19.1
- Intracranial hemorrhage	12	5.1
Atrial fibrillation	111	47
Hypertension	174	73.7
Coronary artery disease	50	21.2
Hypercholesterolemia	135	57.2
Diabetes	49	20.8
- Type I Diabetes mellitus	1	0.4
- Type II Diabetes mellitus	48	20.3
Heart failure	72	30.5
Location of Clot/occlusion		
- ICA, with or without MCA component	37	15.7
- M1 segment	117	49.6
- M2 segment	76	32.2
- M3 segment	6	2.5
Affected hemisphere		
- Left	121	51.3
- Right	115	48.7
Onset time verified	154	65.3
Unknown onset time n (%)	82	34.7

Abbreviations: SD=Standard deviation, TIA=Transient Ischemic Attack, ICA=Internal Carotid Artery, MCA=Middle Cerebral Artery.

**Fig. 1:**
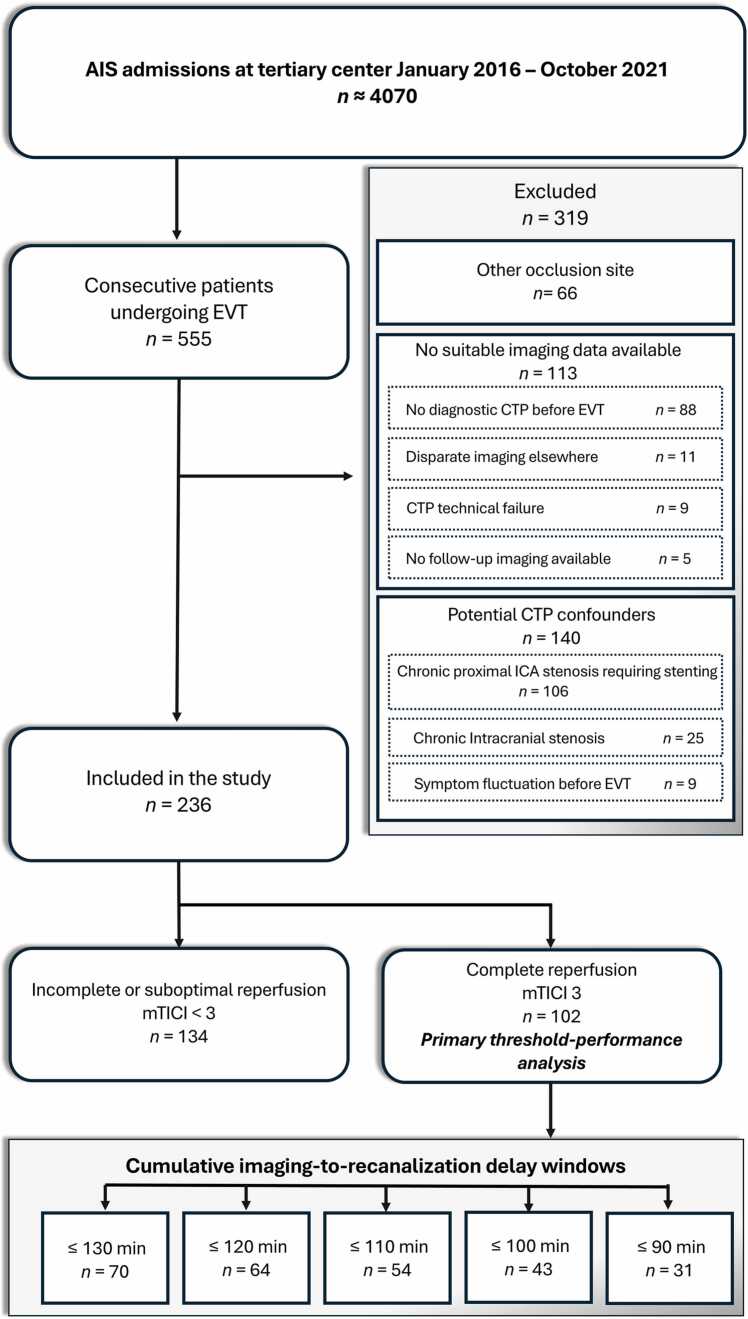
Flow diagram of patient selection and subgroup formation. Among 555 consecutive patients undergoing endovascular treatment (EVT) for acute ischemic stroke (AIS), 236 met the study inclusion criteria for threshold analysis. Exclusions were due to other occlusion sites, absence of technically suitable imaging data, or potential confounders affecting CTP interpretation. To better assess the relationship between admission ischemic core volume (ICV) and follow-up infarct volume (FIV), while minimizing the influence of infarct growth in incompletely recanalized tissue, the primary threshold-performance analysis focused on patients achieving complete reperfusion (mTICI 3, n = 102), with additional cumulative imaging-to-recanalization delay subgrouping at ≤ 130, ≤ 120, ≤ 110, ≤ 100, and ≤ 90 min.

A total of 319 patients were excluded for the following reasons: other occlusion site (n = 66); absence of technically suitable imaging data, including no diagnostic CTP before treatment due to rapid access to EVT (n = 88), CTP performed on a different system elsewhere (n = 11), CTP technical failure (n = 9), or lack of follow-up imaging because of in-hospital death (n = 5); and potential sources of bias in CTP interpretation, including chronic proximal ICA stenosis requiring perioperative carotid artery stenting (n = 106), chronic intracranial stenosis on angiography (n = 25), or symptom fluctuation before EVT (n = 9). The patient selection process is summarized in the flow diagram shown in Fig. 1.

### 2.2 Image acquisition

During admission, all patients underwent a routine stroke imaging protocol, including NECT and cervicocranial CTA, to determine the occlusion site. CTP was performed on SOMATOM Definition Flash or SOMATOM Definition Edge scanners (Siemens Healthcare, Erlangen, Germany) equipped with 64-row Stellar detectors, providing 10 cm brain coverage using sequence shuttle mode (images acquired every 1.5 s, 80 kVp, 80 mAs, 64 × 0.6 mm collimation, 0.57 s, CTDIvol = 84 mGy, total scan time 35 s), using a contrast bolus of 50 mL (Omnipaque 350 mg/mL, 6 mL/s).

Follow-up NECT imaging after EVT was obtained at a median delay of 18 h (IQR 9–65 h) from admission imaging using the SOMATOM Definition Flash scanner. Scans were performed either with a non-contrast helical brain CT protocol with a 64 × 0.6 mm collimation, 100 kVp, quality reference mAs 322, 0.55 s rotation time, pitch 0.55, 4D dose modulation (CAREDose), and organ-based modulation (X-care) enabled, or with dual-energy CT (40 × 0.6 mm collimation, 80 kVp and 140 kVp, ref. mAs 360 and 180, 0.5 s rotation time, pitch 0.55, and 4D dose modulation enabled).

Additional follow-up brain MRI was available in a subset of 44 patients and was used as supportive validation of the NECT measurements. DWI (b1000) obtained during the index hospitalization was available in 23 patients at a median delay of 50 h (IQR 27–71 h), whereas later follow-up T2-weighted imaging was available in an additional 21 patients at a median delay of 3 years (IQR 1–4 years). Imaging was performed using various 1.5-T and 3-T scanners. A detailed list is provided in Supplementary File 1.

### 2.3 Image postprocessing

The CTP images were analyzed using a single software package (syngo.via Neuro Perfusion, version VB80F; Siemens Healthcare GmbH, Erlangen, Germany) with automated motion artifact detection, brain tissue segmentation (from air, bone, and cerebrospinal fluid), and cerebrovascular structure identification. The software uses a delay-independent deconvolution method and interhemispheric comparison to generate maps of cerebral blood flow (CBF), cerebral blood volume (CBV), mean transit time (MTT), time to peak (TTP), time to drain (TTD), and time to maximum (Tmax) with the side showing the longest time to drain designated as the lesion side [26]. Volume estimation maps predicting the ischemic core (IC) and penumbral tissue (i.e., total hypoperfused tissue – IC) are calculated based on these parameters. Developer-recommended thresholds define the IC as areas with a relative CBF (rCBF) < 20% and hypoperfused tissue as areas with Tmax > 6 s, consistent with studies harmonizing results with RAPID [24], [25]. ICV, together with total hypoperfused tissue volume, was calculated from the affected hemisphere using 11 rCBF thresholds ranging from < 10% to < 30% in 2% increments. This range was selected to cover recently proposed threshold values [24], [25], [27] while allowing broader evaluation around them. Smoothing remained enabled throughout all analyses, consistent with earlier comparative work showing improved agreement between syngo.via and RAPID when this setting was applied [23].

Follow-up imaging was reviewed using IDS7 diagnostic workstation (Sectra Imtec 27.1, Linköping, Sweden). Follow-up infarct volume (FIV) was retrospectively assessed primarily on follow-up NECT by a single observer (main author), who manually traced infarcted areas on each consecutive 4–5 mm transverse slice, multiplied each area by slice thickness, and summed the resulting section volumes using Simpson’s method, while blinded to all data except the affected hemisphere and occlusion site [28]. In a supportive validation subset, MRI-based FIV measurements were obtained from either DWI during hospitalization or T2-weighted imaging at later follow-up, using the same multiplication and summation method on 3–5 mm transverse slices, performed by the same observer under otherwise similar blinding conditions.

### 2.4 Endovascular treatment and reperfusion success

Endovascular procedures were performed by experienced neurointerventional radiologists in a dedicated neuroangiography suite (Siemens Healthcare, Erlangen, Germany). A detailed description of the procedures is provided in the Supplementary File 2.

Postprocedural angiographic reperfusion was retrospectively graded by a neurointerventional radiologist (second author) using the modified Treatment in Cerebral Infarction (mTICI) score [29]. In this grading system, mTICI 3 corresponds to complete antegrade reperfusion of the previously occluded target artery ischemic territory without visualized distal branch occlusion. A more detailed description of the classification is provided in Supplementary Table 1**.**

### 2.5 Statistical analysis

Statistical analyses were performed using SPSS for Windows version 29.0.2.0 (IBM Corp., Armonk, NY, USA). Categorical variables are presented as absolute frequencies and percentages. Continuous variables are reported as means ± standard deviation (SD) or medians with interquartile ranges (IQR) for skewed variables. Because the measured values did not follow a normal distribution, as determined by the Kolmogorov–Smirnov test, the Mann–Whitney *U* test was used for group comparisons. Wilcoxon’s signed-rank test was applied to confirm paired differences in ICV obtained with different threshold values.

In subgroup analyses, patients were dichotomized according to reperfusion success (i.e., mTICI 3 vs. other mTICI scores). Among patients achieving complete reperfusion (mTICI 3), additional analyses were performed using cumulative imaging-to-recanalization delay windows defined in 10‑minute increments between 90 and 130 min (i.e., ≤90, ≤100, ≤110, ≤120, and ≤130 min from admission imaging to the time of successful recanalization). Because these delay windows were cumulative, they were not independent, and comparisons across adjacent windows were interpreted descriptively. The subgrouping process is illustrated in Fig. 1**.**

Within each cumulative window, Pearson’s correlation coefficient was calculated to assess the association between admission CTP-derived ICV and NECT-derived FIV across rCBF threshold settings, and the results were visualized using line charts. Ninety-five percent confidence intervals for Pearson’s correlation coefficients were estimated using Fisher’s z transformation. Selected threshold comparisons were further characterized using agreement-oriented measures, including mean bias, 95% limits of agreement, mean absolute error, median FIV − ICV difference, and the frequency and magnitude of ICV overestimation, defined as ICV exceeding FIV. When adjacent thresholds showed similar correlations, preference was given to thresholds with less frequent and smaller-volume overestimation. Bland–Altman plots and scatter plots were used to visualize agreement and the relationship between ICV and FIV. Threshold comparisons were therefore interpreted as exploratory and descriptive rather than as evidence of statistically distinct performance between neighboring thresholds. Statistical significance was set at p < 0.05.

## 3 Results

### 3.1 Baseline data and procedural outcomes

Of the 555 consecutive patients evaluated, 236 were included in this study. A total of 127 (53.8%) patients received intravenous thrombolysis before the procedure. Complete reperfusion (mTICI 3) was achieved in 102 patients, mTICI 2b in 88 patients, and mTICI < 2b in 46 patients. In 154 patients, the symptom onset time could be verified; in this group, the median time from onset to admission imaging delay was 120 min (IQR 71 – 202 min). In the overall study population, median imaging-to-recanalization time was 119 min (IQR 92–166 min). Among patients achieving complete reperfusion, 70 were treated within ≤ 130 min, 64 within ≤ 120 min, 54 within ≤ 110 min, 43 within ≤ 100 min, and 31 within ≤ 90 min from admission imaging. Treatment characteristics and postoperative complications are presented in Table 2**.**

**Table 2:** Procedural, imaging, and outcome characteristics.

**Procedural characteristic**	**n**	**%**	**mean ± SD or** **median (IQR)**
All patients	236	100	
Preoperative IV Thrombolysis	127	53.8	
Preoperative NIHSS mean ± SD	223	94.5	12.9 ± 6.7
Form of sedation/anesthesia			
- Conscious sedation	174	73.7	
- General anesthesia	21	8.9	
- Local anesthesia	41	17.4	
Onset to reperfusion (min) median (IQR)	154	65.3	250.5 (175.8–347.3)
Onset to imaging (min) median (IQR)	154	65.3	120.0 (70.8–202)
Imaging to reperfusion (min) median (IQR)	236	100	119.0 (92.0–166.0)
Intra-arterial procedure			
- Retriever	180	76.3	
- Aspiration	22	9.3	
- IC PTA or permanent stenting	11	4.7	
- Clot not reached	9	3.8	
- No target in angiography	14	5.9	
mTICI score			
- mTICI under 2b	46	19.5	
- mTICI 2b	88	37.3	
- mTICI 3	102	43.2	
Follow-up CT delay (hours) median (IQR)			18 (9–65)
DWI MRI delay (hours) median (IQR)	23	9.7	50 (27–71)
Later MRI delay (years) median (IQR)	21	8.9	3 (1–4)
FIV volume (mL), median (IQR)	236	100	23 (5.4–71.7)
FIV volume (mL) mean ± SD	236	100	60.8 ± 92.7
FIV over 70 mL n (%)	62	26.3	
Total hypoperfused tissue volume (mL) median (IQR)	236	100	111.0 (69.5–161.4)
Total hypoperfused tissue volume (mL) mean ± SD	236	100	123.1 ± 74.1
- Difference from FIV (mL) median (IQR), mL			−67.5 (−110.9–−14.2)
- Difference from FIV (mL) mean ± SD			−62.3 ± 82.7
Postoperative complications	50	21.2	
- Intracerebral hemorrhage	11	4.7	
- Minor sulcal or petechial hemorrhage	21	8.9	
- Death during hospitalization	18	7.6	

Abbreviations: NIHSS=National Institutes of Health Stroke Scale, mTICI=modified Treatment in Cerebral Infarction score, IQR=Interquartile Range, SD=Standard deviation, IC PTA = Intracranial Transluminal Angioplasty, FIV=Follow-up Infarct Volume.

### 3.2 Follow-up infarct volumes

In all patients (n = 236), the median FIV was 23.0 mL (IQR 5.4–71.7 mL), with 62 patients (26.3%) showing a FIV exceeding 70 mL on follow-up NECT. Among patients achieving complete reperfusion mTICI 3 (n = 102), the median FIV was 12.0 mL (IQR 3.3–51.8 mL), and 19 patients (18.6%) had a FIV greater than 70 mL.

The manually traced FIV measurements on follow-up NECT were also evaluated against MRI in a secondary subset. In patients with DWI obtained during hospitalization (n = 23), the median MRI-based FIV was 16.4 mL (IQR 6.6–93.2 mL), showing good correlation with NECT-derived FIV (R = 0.88). In the broader MRI subset, which also included later follow-up T2-weighted imaging (additional n = 21), the combined median MRI-based volume was 15.0 mL (IQR 2.8–58.4 mL), with a correlation of R = 0.86 relative to NECT-derived FIV (n = 44). These MRI comparisons were considered supportive only, given the heterogeneity in MRI timing and sequence type.

### 3.3 Correlation between ICV and FIV

#### 3.3.1 All patients

When ICV was calculated using 11 different threshold levels ranging from rCBF < 10% to < 30% in 2% increments, significant differences were observed both between the resulting ICV estimates and between ICV and FIV volumes (p < 0.01). The relationships between ICV and FIV at selected threshold settings, together with agreement-oriented measures of associated bias, including mean bias, 95% limits of agreement, mean absolute error, and overestimation profile, are summarized in Table 3, whereas more detailed results across all evaluated thresholds are provided in Supplementary Tables 2 and 3**.**

**Table 3:** Summary of selected rCBF thresholds, including highest-correlation thresholds and clinically relevant comparator thresholds, with agreement-oriented metrics relative to follow-up infarct volume.

**Subgroup**	**Highest-correlating** **rCBF threshold**	**n**	**R (95% CI)**	**Mean FIV−ICV (95% LoA), mL**	**MAE (mL)**	**Median FIV−ICV (IQR), mL**	**ICV overestimation, n (%)**	**Median excess in overestimated cases (IQR), mL**
mTICI < 3	< 30%	134	0.553 (0.423–0.661)	42.1 (−113.4–197.5)	50.0	17.1 (−1.0–58.1)	35 (26.1)	7.6 (2.7–15.0)
mTICI 3	< 24%	102	0.777 (0.686–0.844)	33.6 (−103.5–170.8)	37.7	7.8 (0.0–34.9)	25 (24.5)	6.5 (1.8–11.2)
mTICI 3, ≤ 120 min	< 24%	64	0.844 (0.755–0.903)	34.5 (−117.7–186.8)	39.1	7.3 (−0.6–24.9)	18 (28.1)	6.0 (2.5–9.1)
mTICI 3, ≤ 100 min	< 22%*	43	0.863 (0.760–0.924)	35.7 (−131.4–202.8)	39.1	5.1 (0.0–20.6)	9 (20.9)	5.8 (2.0–14.1)
mTICI 3, ≤ 100 min	< 20%*	43	0.863 (0.760–0.924)	38.0 (−133.7–209.7)	40.4	5.5 (0.0–22.0)	8 (18.6)	4.1 (1.3–12.8)

Abbreviations: ICV, ischemic core volume; FIV, follow-up infarct volume; MAE, mean absolute error; mTICI, modified Treatment in Cerebral Infarction; rCBF, relative cerebral blood flow; IQR, interquartile range; LoA, limits of agreement; R, Pearson’s correlation coefficient. Positive FIV−ICV values indicate underestimation of follow-up infarct volume by admission ICV, whereas negative values indicate ICV overestimation. Selected thresholds are shown for the primary and descriptive subgroup comparisons; the full threshold matrix for mTICI 3 patients is provided in Supplementary Tables 2 and 3. All correlations were significant at the 0.01 level. *Correlations were equal at rCBF thresholds of < 22% and < 20% in the ≤ 100-minute subgroup.

In the overall study population (n = 236), the largest ICV estimates were obtained at the rCBF < 30% threshold, yielding a median ICV of 12.6 mL (IQR 4.8–31.9 mL), which also showed the highest observed correlation with FIV (R = 0.61). Estimated ICV decreased progressively toward the more stringent thresholds and approached zero at rCBF < 10%, with a median ICV of 0 mL (IQR 0–1.7 mL; R = 0.52). The rCBF < 30% threshold resulted in ICV overestimation in 76 patients (32.2%; median excess volume 9.5 mL; IQR 3.6–19.3 mL). In contrast, the rCBF < 10% threshold produced ICV overestimation in only 10 patients (4.2%; median excess volume 1.4 mL; IQR 0.5–1.9 mL).

#### 3.3.2 Patients with successful recanalization

Patients without complete reperfusion were excluded from the primary threshold-performance analysis. Within the mTICI 3 subgroup (n = 102), the rCBF < 24% threshold showed the highest observed correlation with FIV (R = 0.78), with a median ICV of 5.4 mL (IQR 1.3 – 15.0 mL). At this threshold, the median FIV−ICV difference was 7.8 mL (IQR 0.0–34.9 mL), and ICV overestimation occurred in 25 patients (24.5%), with a median excess volume of 6.5 mL (IQR 1.8–11.2 mL). Additional agreement-oriented metrics for this threshold are summarized in Table 3. The correlation between ICV and FIV declined gradually toward both higher and lower thresholds, whereas more stringent thresholds were associated with less frequent ICV overestimation.

#### 3.3.3 Subgroups based on admission imaging-to-recanalization delays

**Fig. 2:**
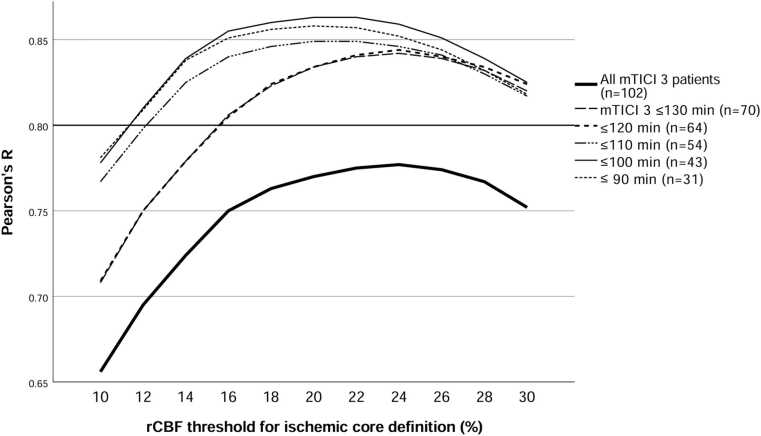
Pearson’s correlation coefficients between ischemic core volumes (ICV) and follow-up infarct volumes (FIV) at each relative cerebral blood flow (rCBF) threshold are shown for patients who achieved full modified Treatment in Cerebral Infarction (mTICI) grade 3 recanalization across different imaging-to-recanalization delay groups. The highest observed correlation (R=0.86) was seen in patients treated within ≤ 100 min using relative rCBF limits of < 22% and < 20% (n = 43). In the broader cumulative subgroup with complete reperfusion within ≤ 120 min, the highest observed correlation with FIV (R=0.84) was seen at the rCBF < 24% threshold (n = 64).

The mTICI 3 subgroup was further examined across cumulative imaging-to-recanalization delay windows. The correlation coefficients between ICV and FIV across the delay subgroups are shown in Fig. 2**.** Across these cumulative windows, the highest observed correlations shifted across adjacent rCBF thresholds, with more stringent thresholds showing slightly higher correlations in the most rapidly reperfused subgroups. Within the cumulative subgroup of patients with complete reperfusion within ≤ 100 min (n = 43), the highest observed correlation with FIV (R = 0.86) was seen at both the rCBF < 22% threshold (median ICV 3.1 mL; IQR 0.4–19.0 mL) and the rCBF < 20% threshold (median ICV 1.5 mL; IQR 0–14.6 mL). Between these two thresholds, rCBF < 20% showed the more favorable overestimation profile, with ICV overestimation in 8 patients (18.6%; median excess 4.1 mL; IQR 1.3–12.8 mL). The overall median imaging-to-recanalization delay was 119 min. In the broader cumulative subgroup of patients with complete reperfusion within ≤ 120 min (n = 64), the highest observed correlation with FIV (R = 0.84) was seen at the slightly less stringent rCBF < 24% threshold. At this threshold, ICV overestimation occurred in 18 patients (28.1%; median excess 6.0 mL; IQR 2.5–9.1 mL). Additional agreement-oriented metrics are summarized in Table 3. At the selected thresholds, mean FIV−ICV bias remained positive and LoA were wide: 33.6 mL (−103.5–170.8) for rCBF < 24% in all mTICI 3 patients, 34.5 mL (−117.7–186.8) for rCBF < 24% in the ≤ 120-minute subgroup, and 38.0 mL (−133.7–209.7) for rCBF < 20% in the ≤ 100-minute subgroup.

**Fig. 3:**
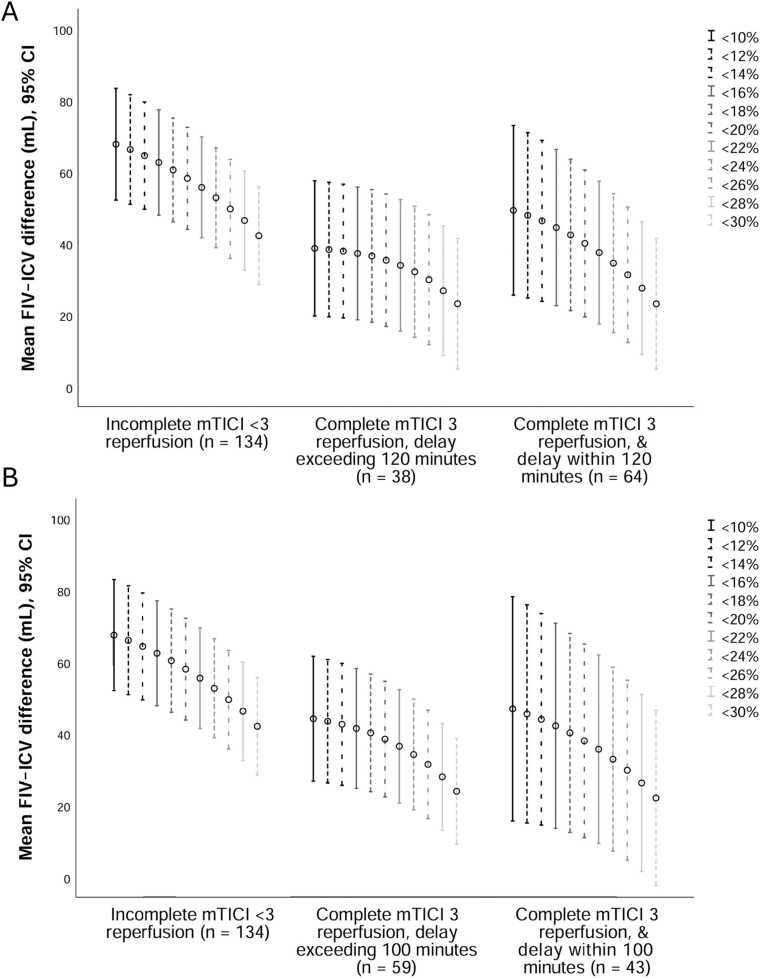
Mean FIV − ICV differences with 95% confidence intervals (CIs) across rCBF thresholds in patients without complete reperfusion and in patients achieving complete reperfusion across cumulative imaging-to-recanalization delay subgroups. A) Comparison of patients without complete reperfusion (n = 134), patients with complete reperfusion exceeding 120 min (n = 38), and patients with complete reperfusion within ≤ 120 min (n = 64). B) Comparison of patients without complete reperfusion (n = 134), patients with complete reperfusion exceeding 100 min (n = 59), and patients with complete reperfusion within ≤ 100 min (n = 43).

**Fig. 4:**
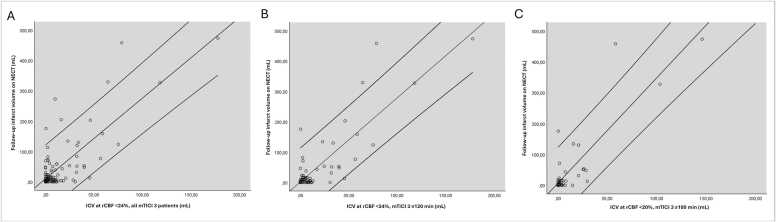
Scatter plots illustrating the relationship between admission ischemic core volume (ICV, mL) and follow-up NECT-derived infarct volume (FIV, mL) in patients achieving complete modified Treatment in Cerebral Infarction (mTICI) grade 3 reperfusion. The linear regression line and its 95% prediction interval are shown in each panel. A) ICV at rCBF < 24% in all mTICI 3 patients (n = 102). B) ICV at rCBF < 24% in the cumulative subgroup with complete reperfusion within ≤ 120 min (n = 64). C) ICV at rCBF < 20% in the cumulative subgroup with complete reperfusion within ≤ 100 min (n = 43).

**Fig. 5:**
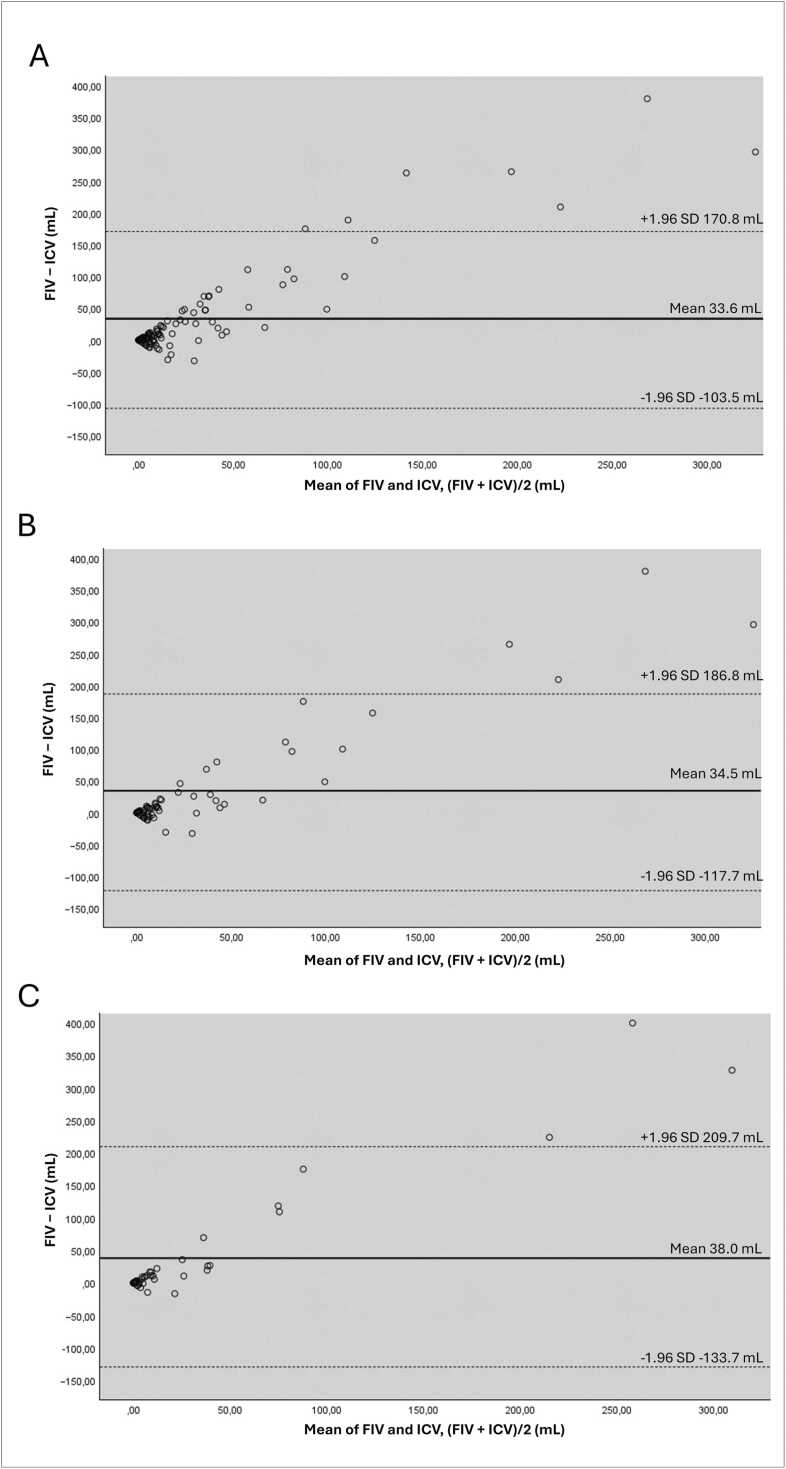
Bland–Altman plots showing agreement between follow-up infarct volume (FIV) and admission ischemic core volume (ICV) in patients who achieved complete modified Treatment in Cerebral Infarction (mTICI) grade 3 reperfusion. The y-axis shows the signed difference between FIV and ICV (FIV − ICV, mL), and the x-axis shows the mean of FIV and ICV [(FIV + ICV)/2, mL]. The center line represents the mean bias, and the outer lines represent the 95% limits of agreement. Negative values indicate ICV overestimation relative to FIV. A) rCBF < 24% in all mTICI 3 patients (n = 102). B) rCBF < 24% in the cumulative subgroup with complete reperfusion within ≤ 120 min (n = 64). C) rCBF < 20% in the cumulative subgroup with complete reperfusion within ≤ 100 min (n = 43).

**Fig. 6:**
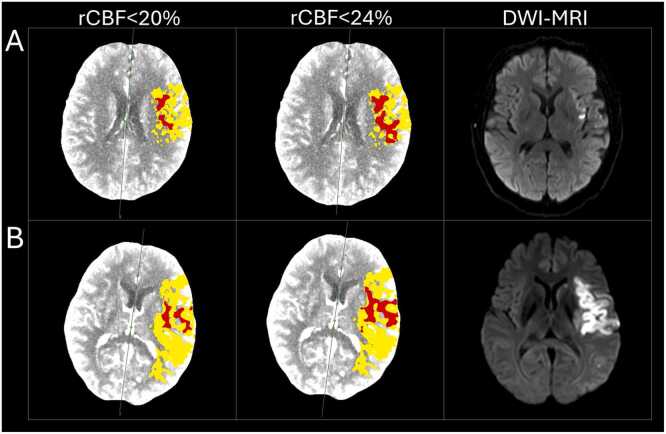
Illustrative cases of two left-sided MCA occlusion strokes with differing imaging-to-recanalization delays, demonstrating variability in admission computed tomography perfusion (CTP)-derived ischemic core volume (ICV) estimates. Irreversibly ischemic tissue is shown in red and hypoperfused penumbral tissue in yellow. A) A 55-year-old male with an M2 level occlusion and an insular infarction on DWI-MRI control imaging, best matching the ICV detected with relative cerebral blood flow (rCBF) < 20% threshold. Imaging-to-recanalization delay was 106 min, achieved reperfusion: mTICI 2b. B) A 49-year-old female with a left-sided MCA M1 occlusion showed an ischemic core estimate that visually corresponded better with the less stringent rCBF < 24% threshold. The imaging-to-recanalization delay was 145 min, and the final reperfusion grade was mTICI 2b. These cases are shown for illustration only and did not meet the mTICI 3 inclusion criterion for the primary threshold-performance analysis.

The most relevant threshold pairs are further illustrated in relation to patients without complete reperfusion (mTICI <3) using mean FIV−ICV differences with corresponding confidence intervals in Fig. 3. Nonparametric comparisons showed the strongest differences in FIV−ICV distributions between patients without complete reperfusion and those achieving rapid complete reperfusion within the ≤ 100- and ≤ 120 min windows; detailed p-values are provided in Supplementary Table 4**.** Scatter plots showed positive ICV–FIV associations in the selected mTICI 3 subgroups (Fig. 4), while Bland–Altman plots showed positive mean FIV−ICV bias with wide 95% limits of agreement, indicating variable individual-level agreement despite high correlations (Fig. 5). Illustrative cases of patients with similar admission infarct profiles but differing imaging-to-recanalization delays are shown in Fig. 6**.** Individual patients also demonstrated non-concordant CTP and FIV results attributable to the so-called ghost infarct core phenomenon [11], [12], [13], and representative examples are shown in Supplementary Fig. 1**.**

## 4 Discussion

In this retrospective single-center study, admission CTP-derived ischemic core volume was compared with follow-up infarct volume across 11 syngo.via rCBF thresholds ranging from < 10% to < 30% in 2% increments. Threshold performance was further examined in patients achieving complete mTICI 3 reperfusion, with imaging-to-recanalization delay evaluated across cumulative windows of ≤ 90, ≤ 100, ≤ 110, ≤ 120, and ≤ 130 min.

A recent study reported that less stringent rCBF thresholds of < 38% for RAPID and < 25% for syngo.via more accurately reflected subsequent NECT-derived FIV in patients achieving complete mTICI 3 reperfusion within 2 h of admission imaging [27]. In the corresponding ≤ 120-minute subgroup of the present study, we observed comparable findings, with the highest observed correlation between admission ICV and FIV at rCBF < 24% (R = 0.84). The same threshold also showed the highest observed correlation in the overall mTICI 3 subgroup, although the correlation was weaker when imaging-to-recanalization delay was not considered (R = 0.78). Across the cumulative delay windows, however, the best-correlating threshold varied, with more stringent rCBF thresholds observed in the more rapidly reperfused subgroups. In patients achieving complete reperfusion within ≤ 100 min, the highest observed correlation was seen at both rCBF < 22% and < 20% (R = 0.86); between these tied thresholds, rCBF < 20% showed a less frequent and smaller-volume overestimation profile, although adjacent thresholds showed very similar correlations.

Although the ≤ 100-minute subgroup yielded the highest observed correlations, agreement-oriented metrics did not identify a single superior threshold. Adjacent rCBF thresholds showed similar correlations but distinct error profiles: more stringent thresholds reduced both the frequency and magnitude of ICV overestimation, whereas slightly less stringent adjacent thresholds produced marginally lower mean FIV−ICV bias and MAE. In the ≤ 100-minute subgroup, rCBF < 20% and < 22% showed comparable correlations but different error profiles: rCBF < 20% was associated with slightly fewer and smaller-volume ICV overestimations, whereas rCBF < 22% showed marginally lower mean FIV−ICV bias and MAE. Scatter plots demonstrated positive ICV–FIV associations in the selected mTICI 3 subgroups, but Bland–Altman analyses showed wide limits of agreement and consistently positive mean FIV−ICV bias, indicating substantial individual-level variability despite strong group-level correlations. The Bland–Altman limits of agreement were interpreted descriptively because the FIV−ICV differences were skewed and appeared wider at larger infarct volumes. Accordingly, threshold choice should be interpreted in relation to the intended purpose of the estimate, including the relative importance placed on avoiding core overestimation versus minimizing overall volumetric error.

Multiple factors influence infarct progression and the severity of perfusion abnormalities on admission imaging, including collateral vessel status and onset-to-imaging delay [30], [31], [32]. The timing of follow-up imaging also represents an additional variable relevant to threshold interpretation [33]. Interhospital transfer and technically complex EVT may prolong imaging-to-recanalization delay. This interval may be further extended by patient-related factors, such as concomitant cervical carotid and intracranial occlusions. In the present study, patients requiring perioperative carotid stenting for chronic ICA stenosis were excluded to reduce confounding from chronic inflow restriction, although this limits the applicability of the findings to tandem-lesion and technically complex EVT workflows.

In another recent study, substantial ICV overestimation was observed only in patients presenting within 90 min of last-known-well using RAPID software; this effect was mitigated by applying a more conservative threshold level [34]. In our setting, the manufacturer-recommended rCBF < 20% threshold shared the highest observed correlation with rCBF < 22% and showed the more favorable overestimation profile in the ≤ 100-minute subgroup, although rCBF < 22% had marginally lower mean FIV−ICV bias and MAE [24], [25]. These findings support rCBF < 20% as a pragmatic reference threshold within this vendor-specific workflow when avoidance of ICV overestimation is prioritized. More broadly, our findings underscore the value of subgroup analyses when interpreting perfusion-derived core estimates in patients with AIS.

Retrospective evaluation of CTP findings in relation to imaging-to-recanalization delay may provide additional insight into the interpretation of vendor-specific perfusion-derived core estimates under different reperfusion-delay conditions. However, the present findings should be regarded as exploratory and methodological, and their clinical applicability requires further validation. Our results are consistent with previous studies assessing the impact of reperfusion delay on ICV estimation [35], [36]. Earlier investigations examining the association between CTP-derived ICV and postprocedural FIV in fully recanalized patients have generally included relatively small to moderate sample sizes, ranging from 31 to 175 patients [12], [18], [30], [34]. In this context, the present findings suggest that threshold behavior may differ under prolonged reperfusion-delay conditions, such as those encountered during interhospital transfer or technically complex EVT procedures.

Several limitations should be acknowledged. First, this was a retrospective single-center study based on a single vendor platform, and the findings should not be generalized directly to other acquisition or post-processing systems. Scanner hardware limited CTP z-axis coverage to 10 cm, which is generally adequate for evaluating the supratentorial anterior-circulation territories included in this study but does not provide whole-brain coverage. Second, the study population was restricted to patients with technically suitable pretreatment CTP, and the primary threshold-performance analysis was centered on patients achieving complete reperfusion, because this provided the clearest setting for comparing admission ICV with follow-up infarct volume. The findings should therefore be interpreted within this imaging-based, fully reperfused study setting. In addition, the analyses were not adjusted for several potentially relevant factors discussed above that may influence infarct evolution and CTP–FIV agreement [29], [30], [31], [32]. Third, the observed correlations were derived from cumulative, partly overlapping, and therefore non-independent delay windows and should be interpreted as exploratory in that context. Fourth, parenchymal swelling and procedure-related hemorrhage may have influenced volumetric assessment on follow-up imaging. In addition, spatial correspondence between admission CTP-derived ischemic core estimates and follow-up infarct lesions was not evaluated. Although NECT- and MRI-derived FIV measurements showed good correlation, MRI was available only in a subset with heterogeneous follow-up timing and sequence types. Therefore, the acute DWI comparison was considered the most relevant ancillary MRI analysis, whereas the broader comparison including late T2 imaging was interpreted cautiously because chronic volume changes may alter lesion volume. These MRI comparisons served as supportive validation of the NECT-derived FIV measurements, although formal reproducibility assessment was not part of the present analysis. Finally, CTP-derived ischemic core estimates should be interpreted with caution, particularly in the early stroke time window, which is associated with an increased risk of core overestimation [11], [12], [13].

## 5 Conclusions

In this vendor-specific retrospective study, the rCBF threshold showing the highest correlation between admission CTP-derived ischemic core volume and follow-up infarct volume shifted across adjacent thresholds as the cumulative imaging-to-recanalization window changed. Lower rCBF thresholds showed the highest observed correlations in patients achieving rapid complete reperfusion, whereas a slightly less stringent adjacent threshold showed the highest observed correlation in the broader ≤ 120-minute subgroup. These findings suggest that reperfusion delay should be considered when interpreting perfusion-derived core estimates, particularly in transfer-dependent or geographically dispersed EVT workflows where imaging-to-recanalization delays may be prolonged. Because the analyses were exploratory and based on overlapping delay windows, validation in larger datasets using non-overlapping or continuous delay analyses is warranted.

## CRediT authorship contribution statement

S.K., M.T., J.H, and R.V. conceptualized the study. M.T. and R.V. contributed to funding acquisition. Data collection was performed by S.K., E.P. and P.J. Data analysis was performed by S.K. R.V., H.M, and J.H. contributed to data interpretation. S.K. Drafted the initial manuscript. S.K. performed the visualization of the data. All authors critically revised the manuscript and approved the definitive version. P.J., M.T., R.V., and H.M. administered and supervised the study.

## CRediT authorship contribution statement

**Ritva Vanninen:** Writing – review & editing, Validation, Supervision, Project administration, Methodology, Investigation, Funding acquisition, Conceptualization. **Pekka Jäkälä:** Writing – review & editing, Supervision, Resources, Project administration, Data curation. **Mikko Taina:** Writing – review & editing, Supervision, Project administration, Funding acquisition, Conceptualization. **Juhana Hakumäki:** Writing – review & editing, Validation, Supervision, Project administration, Methodology, Investigation, Funding acquisition, Conceptualization. **Hannu Manninen:** Writing – review & editing, Validation, Supervision, Project administration, Methodology, Investigation, Formal analysis. **Erik Palm:** Writing – review & editing, Investigation, Data curation. **Simo Karhi:** Writing – review & editing, Writing – original draft, Visualization, Software, Methodology, Investigation, Formal analysis, Data curation, Conceptualization.

## Ethical statement

The study was conducted according to the principles of the Declaration of Helsinki and was approved by the Research Ethics Board of Kuopio University Hospital (no. 5772789, 12.3.2013). The requirement for informed consent was waived due to the retrospective nature of the study and the use of routinely collected clinical data. Data were analyzed in pseudonymized form in accordance with applicable hospital guidelines and relevant data protection laws and regulations.

## Declaration of Generative AI and AI-assisted technologies in the writing process

During the preparation of this work the authors used ChatGPT (OpenAI) to assist with language editing. After using this tool/service, the authors reviewed and edited the content as needed and take full responsibility for the content of the published article.

## Funding

This study was supported by governmental funding from the Kuopio University Hospital Research Commission (grant number: 5772789). The first author has also received a personal grant from the private Mauri and Sirkka Wiljasalo Testament Fund. Funders did not play any role in the study design, data collection and analysis, decision to publish, or preparation of the manuscript. No additional external funding was received for this study.

## Declaration of Competing Interest

The authors declare that they have no known competing financial interests or personal relationships that could have appeared to influence the work reported in this paper.

## Data Availability

The data underlying the findings of this study includes sensitive and confidential patient information and cannot be shared publicly due to institutional policies of Kuopio University Hospital and applicable Finnish national data protection regulations. It is available from the Kuopio University Hospital / Institute of Clinical Medicine Ethics Committee (contact via corresponding author) upon reasonable requests for researchers who meet the criteria for access to confidential data.
