# Supplementary material for: Imaging-to-recanalization delay influences perfusion CT threshold calibration for follow-up infarct volume estimation
Source: Eur J Radiol Open. 2026 Jun 18;17:100779. doi: 10.1016/j.ejro.2026.100779 (PMC13311185; doi:10.1016/j.ejro.2026.100779)
Supplement: Supplementary file 1 — Supplementary material [file mmc1.docx]

**Supplementary File 1. MRI scanners and software used for follow-up imaging**

Follow-up imaging of the included patients was performed using various scanners and software: Siemens Magnetom AVANTOFit, AERA, Sola, or Vida using syngo MR B19, E11, XA11 or XA20 or XA30 software (Siemens Healthcare, GmbH, Erlangen, Germany); Philips Healthcare Achieva using version 5.3.0.3 software (Philips Healthcare, ORL, USA); Canon Titan using MR software version V4.0SP0037* or 6.0SP6000* (Canon Healthcare, Ōtawara, Japan); or GE Signa using version HD23.0_V03_1614.b software (GE Healthcare, Chicago, IL, USA).
