# Supplementary material for: Imaging-to-recanalization delay influences perfusion CT threshold calibration for follow-up infarct volume estimation
Source: Eur J Radiol Open. 2026 Jun 18;17:100779. doi: 10.1016/j.ejro.2026.100779 (PMC13311185; doi:10.1016/j.ejro.2026.100779)
Supplement: Supplementary file 2 — Supplementary material [file mmc2.docx]

**Supplementary File 2. Description of endovascular procedures**

Endovascular procedures were performed by experienced neurointerventional radiologists in a dedicated neuroangio suite (Siemens Healthcare, Erlangen, Germany) under conscious sedation (N=174), general anaesthesia (N=21) or local anaesthesia (N=41). A multipurpose guiding catheter or balloon guide (Flow Gate; Stryker, Fremont, CA, USA) was taken to internal carotid artery over a 5 Fr diagnostic catheter (JR4; Cordis, Miami, FL, USA) via femoral access with 8F/80 cm introducer (Cook Incorporated, Bloomington, IN, USA). The occlusion was traversed with a microcatheter (Neuroslider 21; Acandis GmbH, Pforzheim, Germany) with the help of a 0.014-inch microwire (Synchro2 or Transend EX; Stryker). An aspiration catheter (5MAX, 5MAX60, or ACE68; Penumbra Inc., Alameda, CA, USA, or React 68; Medtronic, Minneapolis, MN, USA) was further delivered to the target occlusion site over the microcatheter. Thrombus extraction was performed mainly using a retriever (N=180) (Aperio; Acandis or Embotrap; Cerenovus, Irvine, CA, USA), direct aspiration (N=22) and in some cases intracranial percutaneous transluminal angioplasty with or without permanent stenting (N=11). In 9 cases thrombus site was not reached and in 14 cases the clot had already resolved prior to angiography likely due to concomitant thrombolysis treatment. Modified Treatment in Cerebral Infarction score (mTICI) score was retrospectively assessed by a neurointerventional radiologist and was used to evaluate the treatment success. Complete recanalization was defined as mTICI 3 flow.
