# Supplementary material for: Imaging-to-recanalization delay influences perfusion CT threshold calibration for follow-up infarct volume estimation
Source: Eur J Radiol Open. 2026 Jun 18;17:100779. doi: 10.1016/j.ejro.2026.100779 (PMC13311185; doi:10.1016/j.ejro.2026.100779)
Supplement: Supplementary file 3 — Supplementary material [file mmc3.docx]

**Supplementary Table 1: Modified Treatment in Cerebral Infarction (mTICI) score classification (according to Zaidat et al., 2013)**

| **Score** | **Definition** |
| --- | --- |
| mTICI 0 | No perfusion |
| mTICI 1 | Antegrade reperfusion past the initial occlusion, but limited distal branch filling with little or slow distal reperfusion. |
| mTICI 2a | Antegrade reperfusion of less than half of the previously occluded target artery ischemic territory. |
| mTICI 2b | Antegrade reperfusion of more than half of the previously occluded target artery ischemic territory. |
| mTICI 3 | Complete antegrade reperfusion of the previously occluded target artery ischemic territory, with no visualized occlusion in all distal branches. |
|  |  |

O.O. Zaidat, A.J. Yoo, P. Khatri, T.A. Tomsick, R. von Kummer, J.L. Saver, M.P. Marks, S. Prabhakaran, D.F. Kallmes, B.-F.M. Fitzsimmons, J. Mocco, J.M. Wardlaw, S.L. Barnwell, T.G. Jovin, I. Linfante, A.H. Siddiqui, M.J. Alexander, J.A. Hirsch, M. Wintermark, G. Albers, H.H. Woo, D.V. Heck, M. Lev, R. Aviv, W. Hacke, S. Warach, J. Broderick, C.P. Derdeyn, A. Furlan, R.G. Nogueira, D.R. Yavagal, M. Goyal, A.M. Demchuk, M. Bendszus, D.S. Liebeskind, Recommendations on angiographic revascularization grading standards for acute ischemic stroke: a consensus statement, Stroke 44 (2013) 2650–2663. https://doi.org/10.1161/STROKEAHA.113.001972
