# Supplementary material for: Imaging-to-recanalization delay influences perfusion CT threshold calibration for follow-up infarct volume estimation
Source: Eur J Radiol Open. 2026 Jun 18;17:100779. doi: 10.1016/j.ejro.2026.100779 (PMC13311185; doi:10.1016/j.ejro.2026.100779)
Supplement: Supplementary file 4 — Supplementary material [file mmc4.docx]

**Supplementary Table 2. Pearson’s Correlations Between ICV and FIV in Different Recanalization Delay Groups**

| **Threshold level** | **All mTICI 3 patients  (n=102)** | **Imaging to mTICI 3 reperfusion ≤130 min (n=70)** | **Imaging to mTICI 3 reperfusion ≤120 min  (n=64)** | **Imaging to mTICI 3 reperfusion ≤110 min  (n=54)** | **Imaging to mTICI 3 reperfusion ≤100 min  (n=43)** | **Imaging to mTICI 3 reperfusion ≤90 min  (n=31)** |
| --- | --- | --- | --- | --- | --- | --- |
| rCBF <30 % | .752 | .820 | .824 | .817 | .825 | .818 |
| rCBF <28 % | .767 | .832 | .834 | .830 | .839 | .832 |
| rCBF <26 % | .774 | .839 | .840 | .841 | .851 | .844 |
| rCBF <24 % | .777 | .842 | .844 | .846 | .859 | .852 |
| rCBF <22 % | .775 | .840 | .841 | .849 | .863 | .857 |
| rCBF <20 % | .770 | .834 | .834 | .849 | .863 | .858 |
| rCBF <18 % | .763 | .823 | .824 | .846 | .860 | .856 |
| rCBF <16 % | .750 | .806 | .805 | .840 | .855 | .851 |
| rCBF <14 % | .724 | .779 | .779 | .825 | .839 | .838 |
| rCBF <12 % | .695 | .750 | .750 | .798 | .810 | .809 |
| rCBF <10 % | .656 | .708 | .709 | .767 | .778 | .781 |

Abbreviations: ICV= Ischemic core volume, FIV = Follow-up infarct volume, rCBF=relative Cerebral Blood Flow, mTICI=modified Treatment in Cerebral Infarction score, Pearson's R = Pearson's correlation coefficient (All correlations were significant at the 0.01 level).
