# Supplementary material for: Imaging-to-recanalization delay influences perfusion CT threshold calibration for follow-up infarct volume estimation
Source: Eur J Radiol Open. 2026 Jun 18;17:100779. doi: 10.1016/j.ejro.2026.100779 (PMC13311185; doi:10.1016/j.ejro.2026.100779)
Supplement: Supplementary file 5 — Supplementary material [file mmc5.docx]

**Supplementary Table 3A. ICV correlation to FIV in most relevant delay subgroups**

|  |  | **rCBF <30 %** | **rCBF <28 %** | **rCBF <26 %** | **rCBF <24 %** | **rCBF <22 %** | **rCBF <20 %** | **rCBF <18 %** |
| --- | --- | --- | --- | --- | --- | --- | --- | --- |
| **All mTICI 3 patients (n =102)** | **Pearson’s R between ICV and FIV** | .752 | .767 | .774 | .777 | .775 | .770 | .763 |
|  | **FIV - ICV (mL)  median (IQR)** | 1.1 (-9.0–28.2) | 3.2 (-4.4–31.6) | 5.6 (2.1–32.9) | 7.8 (0.0–34.9) | 9.1 (0.1–36.6) | 10.4 (1.0–39.7) | 10.4 (1.4–47.3) |
|  | **Overestimation n (%), median (IQR), (mL)** | 41 (40.2) 11.6 (3.9–20.5) | 33 (32.4) 8.2 (4.4–16.9) | 27 (26.5) 8.1 (2.8–13.7) | 25 (24.5) 6.5 (1.8–11.2) | 18 (17.6) 6.0 (3.6–9.3) | 17 (16.7) 4.1 (1.7–7.4) | 15 (14.7) 2.6 (0.7–5.5) |
| **Recanalization within 120 minutes (n=64)** | **Pearson’s R between ICV and FIV** | .824 | .834 | .840 | .844 | .841 | .834 | .824 |
|  | **FIV - ICV (mL)  median (IQR)** | 0.6 (-11.6–13.8) | 2.2 (-5.8–17.1) | 5.0 (-2.9–21.3) | 7.3 (-0.6–24.9) | 8.2 (0.0–30.8) | 9.1 (0.2–34.8) | 9.7 (0.4–37.2) |
|  | **Overestimation n (%) median (IQR), (mL)** | 28 (43.8) 11.7 (6.7–20.0) | 23 (35.9) 8.2 (5.0–16.0) | 20 (31.3) 7.7 (3.0–13.2) | 18 (28.1) 6.0 (2.5–9.1) | 14 (21.9) 5.8 (3.1–6.9) | 13 (20.3) 3.8 (1.5–6.5) | 11 (17.2) 2.6 (0.7–5.5) |
| **Recanalization within 100 minutes (n=43)** | **Pearson’s R between ICV and FIV** | .825 | .839 | .851 | .859 | .863 | .863 | .860 |
|  | **FIV - ICV (mL)  median (IQR)** | 0.4 (-11.6–7.4) | 1.4 (-5.0–10.9) | 3.0 (-2.8–14.1) | 3.4 (-0.5–15.1) | 5.1 (0.0–20.6) | 5.5 (0.0–22.0) | 6.5 (0.3–25.5) |
|  | **Overestimation n (%), median (IQR), (mL)** | 19 (44.2) 11.6 (4.1–20.8) | 16 (37.2) 7.7 (4.4–16.0) | 13 (30.2) 7.2 (2.9–13.5) | 12 (27.9) 5.1 (1.0–11.5) | 9 (20.9) 5.8 (2.0–14.1) | 8 (18.6) 4.1 (1.3–12.8) | 6 (14.0) 3.9 (1.5–8.9) |

Abbreviations: ICV= ischemic core volume, FIV = Follow-up infarct volume, mTICI=modified Treatment in Cerebral Infarction score rCBF=relative Cerebral Blood Flow, IQR=Interquartile Range, Pearson's R = Pearson's correlation coefficient (All correlations were significant at the 0.01 level)

**Supplementary Table 3B. ICV correlation to FIV in all patients**

|  | **rCBF limit (%)** | **<30** | **<28** | **<26** | **<24** | **<22** | **<20** | **<18** | **<16** | **<14** | **<12** | **<10** |
| --- | --- | --- | --- | --- | --- | --- | --- | --- | --- | --- | --- | --- |
| **All patients (n =236)** | Pearson’s R between ICV and FIV | **.605** | .597 | .598 | .592 | .583 | .574 | .566 | .557 | .545 | .532 | .523 |
|  | ICV (mL) median (IQR) | 12.6 (4.8–31.9) | 9.6 (3.1–24.3) | 7.1 (2.0–20.0) | 5.4 (1.3–14.9) | 3.8 (0.0–11.2) | 2.3 (0.0–8.9) | 1.5 (0.0–6.9) | 1.0 (0.0–4.7) | 0.0 (0.0–3.8) | 0.0 (0.0–2.7) | 0.0 (0.0–1.7) |
|  | FIV - ICV (mL) median (IQR) | 7.9 (-3.1–41.4) | 10.6 (-0.4–43.2) | 12.1 (0.0–48.0) | 14.1 (1.4–51.7) | 16.1 (2.3–57.2) | 17.5 (2.8–61.0) | 18.7 (3.2–65.9) | 19.9 (3.5–68.4) | 20.7 (4.0–69.1) | 21.7 (4.4–69.1) | 21.8 (5.1–70.5) |
|  | ICV Overestimation (mL) median (IQR), n | 9.5 (3.6-19.3), 76 | 7.3 (4.4–15.9), 60 | 5.7 (2.7–12.6), 50 | 5.0 (1.7–8.8), 42 | 5.2 (2.4–8.2), 32 | 3.4 (1.5–6.1), 30 | 2.3 (1.1–4.6), 26 | 1.9 (1.2–3.5), 19 | 1.3 (0.9–2.9), 14 | 1.5 (0.8–2.7), 11 | 1.4 (0.5–1.9), 10 |
| **All mTICI 3 patients (n =102)** | Pearson’s R between ICV and FIV | .752 | .767 | .774 | **.777** | .775 | .770 | .763 | .750 | .724 | .695 | .656 |
|  | ICV (mL) median (IQR) | 12.6 (3.9–32.2) | 9.6 (3.1–24.3) | 7.1 (2.0–20.0) | 5.4 (1.3–15.0) | 3.8 (0.0–14.9) | 2.3 (0.0–8.9) | 1.5 (0.0–8.9) | 1.5 (0.0–6.9) | 1.0 (0.0–4.7) | 0.0 (0.0–2.7) | 0.0 (0.0–1.7) |
|  | FIV - ICV (mL) median (IQR) | 1.1 (-9.0–28.2) | 3.2 (-4.4–31.6) | 5.6 (2.1–32.9) | 7.8 (0.0–34.9) | 9.1 (0.1–36.6) | 10.4 (1.0–39.7) | 10.4 (1.4–47.3) | 10.4 (2.0–48.0) | 10.4 (2.0–48.0) | 10.4 (2.1–48.5) | 10.4 (2.8–49.2) |
|  | ICV Overestimation, (mL) median (IQR), n | 11.6 (3.9–20.5), 41 | 8.2 (4.4–16.9), 33 | 8.1 (2.8–13.7), 27 | 6.5 (1.8–11.2), 25 | 6.0 (3.6–9.3), 18 | 4.1 (1.7–7.4), 17 | 2.6 (0.7–5.5), 15 | 2.6 (1.1–3.7), 10 | 2.0 (0.8–3.2), 6 | 2.1 (0.7–3.1), 4 | 1.4 (*–2.6), 3 |
| **mTICI 3 within 120 minutes (n=64)** | Pearson’s R between ICV and FIV | .824 | .834 | .840 | **.844** | .841 | .834 | .824 | .805 | .779 | .750 | .709 |
|  | ICV (mL) median (IQR) | 13.1 (5.7–43.2) | 9.7 (3.9–36.8) | 7.3 (2.5–29.6) | 5.8 (1.6–24.2) | 4.6 (1.1–19.1) | 2.9 (0.5–15.2) | 1.9 (0.0–8.5) | 1.4 (0.0–7.2) | 0.7 (0.0–6.1) | 0.0 (0.0–3.9) | 0.0 (0.0–2.3) |
|  | FIV - ICV (mL) median (IQR) | 0.6 (-11.6–13.8) | 2.2 (-5.8–17.1) | 5.0 (-2.9–21.3) | 7.3 (-0.6–24.9) | 8.2 (0.0–30.8) | 9.1 (0.2–34.8) | 9.7 (0.4–37.2) | 10.3 (0.4–39.9) | 10.3 (1.3–41.9) | 10.3 (1.5–44.6) | 10.3 (1.5–45.4) |
|  | ICV Overestimation (mL) median (IQR) | 11.7 (6.7–20.0), 28 | 8.2 (5.0–16.0), 23 | 7.7 (3.0–13.2), 20 | 6.0 (2.5–9.1), 18 | 5.8 (3.1–6.9), 14 | 3.8 (1.5–6.5), 13 | 2.6 (0.7–5.5), 11 | 2.3 (0.8–3.8), 9 | 2.9(1.0–3.6), 5 | 2.1 (0.7–3.1), 4 | 1.5 (*–2.6), 3 |
| **mTICI 3 within 100 minutes (n=43)** | Pearson’s R between ICV and FIV | .825 | .839 | .851 | .859 | **.863** | **.863** | .860 | .855 | .839 | .810 | .779 |
|  | ICV (mL) median (IQR) | 9.4 (3.4–39.8) | 7.7 (2.6–34.3) | 6.1 (1.5–29.3) | 4.8 (0.5–23.0) | 3.1 (0.4–19.0) | 1.5 (0.0–14.6) | 1.4 (0.0–8.7) | 0.4 (0.0–6.6) | 0.0 (0.0–6.1) | 0.0 (0.0–3.3) | 0.0 (0.0–1.6) |
|  | FIV - ICV (mL) median (IQR) | 0.4 (-11.6–7.4) | 1.4 (-5.0–10.9) | 3.0 (-2.8–14.1) | 3.4 (-0.5–15.1) | 5.1 (0.0–20.6) | 5.5 (0.0–22.0) | 6.5 (0.3–25.5) | 7.1 (0.4–31.4) | 7.4 (1.1–35.3) | 7.7 (1.1–38.1) | 8.0 (1.1–42.2) |
|  | ICV Overestimation (mL) median (IQR), n | 11.6 (4.1–20.8), 19 | 7.7 (4.4–16.0), 16 | 7.2 (2.9–13.5), 13 | 5.1 (1.0–11.5), 12 | 5.8 (2.0–14.1), 9 | 4.1(1.3–12.8), 8 | 3.9 (1.5–8.9), 6 | 3.2 (1.7–5.8), 4 | 3.6(*–4.3), 2 | 2.4 (*–3.2), 2 | 1.4 (*–1.5), 2 |

Abbreviations: ICV= ischemic core volume, FIV = Follow-up infarct volume, mTICI=modified Treatment in Cerebral Infarction score rCBF=relative Cerebral Blood Flow, IQR=Interquartile Range, Pearson's R = Pearson's correlation coefficient (All correlations were significant at the 0.01 level). *IQR not reported due to small n.
