# Supplementary material for: Imaging-to-recanalization delay influences perfusion CT threshold calibration for follow-up infarct volume estimation
Source: Eur J Radiol Open. 2026 Jun 18;17:100779. doi: 10.1016/j.ejro.2026.100779 (PMC13311185; doi:10.1016/j.ejro.2026.100779)
Supplement: Supplementary file 6 — Supplementary material [file mmc6.docx]

**Supplementary Table 4. Mann–Whitney U test p-values for between-group comparisons of FIV−ICV differences across selected rCBF thresholds.**

| **rCBF threshold** | **mTICI 3 >120 vs ≤120 min** | **mTICI 3 >100 vs ≤100 min** | **mTICI <3 vs mTICI 3** | **mTICI <3 vs mTICI 3 ≤100 min** | **mTICI <3 vs mTICI 3 >100 min** | **mTICI <3 vs mTICI 3 ≤120 min** | **mTICI <3 vs mTICI 3 >120 min** |
| --- | --- | --- | --- | --- | --- | --- | --- |
| **<10%** | 0.1087 | 0.1087 | 0.0020 | 0.0018 | 0.0539 | 0.0039 | 0.0595 |
| **<12%** | 0.1148 | 0.1148 | 0.0017 | 0.0015 | 0.0479 | 0.0032 | 0.0585 |
| **<14%** | 0.1246 | 0.1246 | 0.0013 | 0.0013 | 0.0408 | 0.0025 | 0.0533 |
| **<16%** | 0.3324 | 0.1350 | 0.0011 | 0.0010 | 0.0398 | 0.0018 | 0.0576 |
| **<18%** | 0.3608 | 0.1441 | 0.0009 | 0.0007 | 0.0417 | 0.0016 | 0.0561 |
| **<20%** | 0.3536 | 0.1368 | 0.0010 | 0.0007 | 0.0440 | 0.0014 | 0.0632 |
| **<22%** | 0.3188 | 0.1351 | 0.0012 | 0.0007 | 0.0529 | 0.0013 | 0.0888 |
| **<24%** | 0.2833 | 0.1248 | 0.0011 | 0.0007 | 0.0509 | 0.0010 | 0.1013 |
| **<26%** | 0.2128 | 0.1003 | 0.0014 | 0.0008 | 0.0601 | 0.0010 | 0.1362 |
| **<28%** | 0.2232 | 0.1316 | 0.0013 | 0.0007 | 0.0564 | 0.0008 | 0.1402 |
| **<30%** | 0.2393 | 0.1944 | 0.0008 | 0.0006 | 0.0374 | 0.0006 | 0.1135 |

Abbreviations: FIV, follow-up infarct volume; ICV, ischemic core volume; rCBF, relative cerebral blood flow; mTICI, modified Treatment in Cerebral Infarction score. Lower p-values indicate stronger evidence of between-group differences in FIV−ICV distributions. Exact p-values are shown descriptively; no adjustment for multiple comparisons was applied. Thresholds are ordered from more stringent to less stringent settings to match the manuscript table sequence. Red shading indicates p < 0.01, yellow shading 0.01 ≤ p < 0.05, and green shading p ≥ 0.05. Comparisons included mTICI 3 subgroup splits at 100 and 120 minutes and subgroup contrasts with patients without complete reperfusion (mTICI <3).
