# Supplementary figures and images for: Imaging-to-recanalization delay influences perfusion CT threshold calibration for follow-up infarct volume estimation
Source: Eur J Radiol Open. 2026 Jun 18;17:100779. doi: 10.1016/j.ejro.2026.100779 (PMC13311185; doi:10.1016/j.ejro.2026.100779)

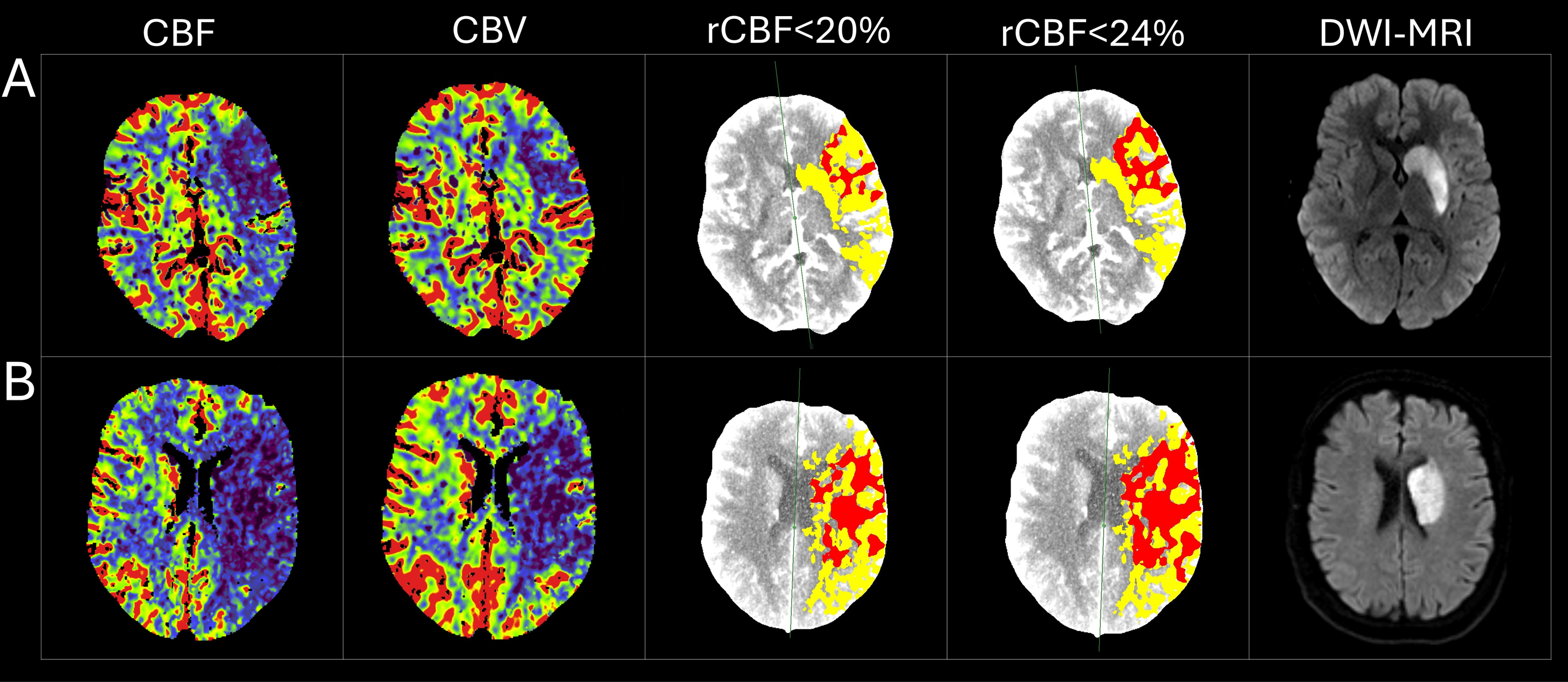

Supplement: Supplementary file 7 — Supplementary material [file mmc7.jpg]
